# Erroneous saccade co-execution during manual action control is independent of oculomotor stimulus-response translation ease

**DOI:** 10.1007/s00426-024-01989-y

**Published:** 2024-07-30

**Authors:** Jens Kürten, Tim Raettig, Lynn Huestegge

**Affiliations:** Department of Psychology, University of Würzburg, Röntgenring 11, 97070 Würzburg, Germany

**Keywords:** Multiple action control, Peripheral stimuli, Symbolic stimuli, Automaticity, Dual-action benefits

## Abstract

Recent multiple action control studies have demonstrated difficulties with single-action (vs. dual-action) execution when accompanied by the requirement to inhibit a prepotent additional response (e.g., a highly automatic eye movement). Such a dual-action performance benefit is typically characterized by frequent false-positive executions of the currently unwarranted response. Here, we investigated whether the frequency of false-positive saccades is affected by the ease of translating a stimulus into a spatial oculomotor response (S-R translation ease): Is it harder to inhibit a saccade that is more automatically triggered via the stimulus? Participants switched on a trial-by-trial basis between executing a single saccade, a single manual button press, and a saccadic-manual dual action in response to a single visual stimulus. Importantly, we employed three different stimulus modes that varied in oculomotor S-R translation ease (peripheral square > central arrow > central shape). The hierarchy of S-R translation ease was reflected by increasing saccade and manual reaction times. Critically, however, the frequency of false-positive saccades in single manual trials was not substantially affected by the stimulus mode. Our results rule out explanations related to limited capacity sharing (between inhibitory control and S-R translation demands) as well as accounts related to the time available for the completion of saccade inhibition. Instead, the findings suggest that the erroneous co-activation of the oculomotor system was elicited by the mere execution of a (frequently associated) manual response (action-based co-activation).

**Supplementary Information:**

The online version contains supplementary material available at 10.1007/s00426-024-01989-y.

Coordinating multiple actions can be challenging compared to concentrating on only one action at a time. A plethora of research has been dedicated to the difficulties associated with simultaneous *execution* of two actions (vs. only one), which can be observed in terms of slower and more error-prone responses (i.e., dual action *costs*, e.g., Fagot & Pashler, 1992; Koch et al., 2018; Pashler, 1994). Some studies, however, have also demonstrated difficulties associated with the overt execution of only a single action, namely in situations when this is accompanied by a requirement for *simultaneous inhibition* of another, highly prepotent action (e.g., Miller, 2006). In these situations, executing both actions turns out to be easier due to the absence of inhibitory demands (dual-action performance *benefit)*. This benefit is often most pronounced in the error data due to frequent false-positive executions of a prepotent second response in single-action trials. The present study investigated how the occurrence of false-positive action executions in single-action trials is affected by stimulus-to-response (S-R) translation ease for the to-be-inhibited response, a factor that up to now has not received systematic empirical attention.

Concurrent executive and inhibitory response demands, giving rise to dual-action benefits, have been studied in variations of the single-dual-switch (SDS) paradigm (see e.g., Huestegge et al., 2023; Huestegge & Strobach, 2021; Strobach & Huestegge, 2021). In the SDS paradigm, participants are required to frequently switch between executing single actions and dual actions, thereby ensuring a high baseline activation of each action type across a block of trials (Lussier et al., 2012; Schumacher et al., 2001). In particular, dual-action benefits are typically observed in a variant of this paradigm involving a single stimulus triggering both single actions and dual actions (see Fagot & Pashler, 1992). Specifically, a cue typically indicates the type of action required (i.e., either a single action [A], the other single action [B], or the combined dual action [A + B]), while a separate target stimulus (or stimulus dimension) indicates the full specification (e.g., direction) of the currently relevant action(s). The requirement to execute only single action [A] can create an (implicit) inhibitory demand (inhibiting the other action [B]) whenever some preconditions are met that affect the urge to execute the currently unwarranted action [B], that is, its *action prepotency*. The typical finding is a preponderance of false-positive executions of the currently unwarranted, but prepotent response alternative along with the required response in single-action trials (Huestegge & Koch, 2014; Raettig & Huestegge, 2018). Inhibitory failures of this kind have been demonstrated for saccadic eye movements combined with manual key presses (Huestegge & Koch, 2014), and for manual key presses combined with directional vocal responses (Raettig & Huestegge, 2018). This might indicate similar mechanisms underlying involuntary co-execution with different response modality combinations, although this remains to be tested more explicitly in the future. Dual-action trials, in contrast, are usually less error-prone due to the absence of inhibitory demands. Sometimes, even faster dual-action (vs. single-action) reaction times (RTs) have been observed, in particular when presenting cue (action type) and imperative stimulus (action direction) as a single compound (Raettig & Huestegge, 2021, 2023).

The generation of inhibitory failures in the SDS paradigm is hard to explain with some traditional models of multiple action control assuming separate (serial or parallel) selection processes for each individual action without reference to content-based interaction between processing streams (Meyer & Kieras, 1997; Navon & Miller, 2002; Pashler, 1994). In contrast, models incorporating the notion of information crosstalk as a central mechanism can more readily explain the phenomenon of unwanted action co-execution (Huestegge, 2011; Logan & Gordon, 2001; Navon & Miller, 1987). In one such model, action selection is achieved by binding the correct action-relevant representations (codes) in working memory during a *unitary* central mapping selection stage (Huestegge, 2011; Huestegge & Koch, 2010) in which all currently relevant responses are prepared. For example, the specification of a manual-oculomotor dual action to the left would require binding (at least) a “left” (vs. “right”) spatial code to both a “manual” and a “saccade” modality code. In contrast, a single manual action to the left would require binding the “left” code only to the “manual” (not the “saccade”) code. In this case, activation could easily spread to the saccade code due to its strong a-priori activation (assuming a generally high prepotency for oculomotor responses, see below), thereby erroneously binding this response modality (code) into the action plan. Correct single-manual execution would therefore require some degree of active inhibition or downregulation of the prepotent saccade modality code (or its association with the spatial codes, see Huestegge & Koch, 2014).

Previous research has established that the likelihood of inhibitory failures (e.g., false-positive saccades) indeed depends to a large degree on the a priori activation of the to-be-inhibited response, that is, its *prepotency* (Ridderinkhof et al., 2014). A number of factors were shown to affect action prepotency. For example, it has been demonstrated that the erroneous execution of an unwarranted response is more likely when this very response was recently required, that is, it is especially difficult to withhold a saccade or manual response in a current trial when saccade or manual response execution was required in the previous trial. Importantly, this phenomenon even occurred irrespective of its particular direction (Kürten et al., 2022, 2023), suggesting that prepotency can be regulated at the level of the whole effector system (e.g., “oculomotor system”), not only at the level of the individual (fully specified) response (e.g., a “saccade of a specific length to the left”). On the other hand, the lack of any requirement to execute the to-be-inhibited response throughout a block of trials renders saccadic inhibition comparatively easy, but, notably, still far from perfect – while manual inhibition almost always succeeds in such a condition (Raettig & Huestegge, 2023). This indicates that some effector systems are inherently more prone to unwarranted execution, such as oculomotor responses compared with manual (or vocal) responses (Kürten et al., 2022). Further, pre-knowledge of the relevant response modality (modalities) allows for pro-active inhibition of unwarranted effector systems given sufficient time, even without pre-knowledge of the required response direction (Kürten et al., 2023; see also Greenhouse et al., 2012). Taken together, these observations show that action prepotency can be dynamically modulated. The present study asked whether a particularly easy (more automatic) S-R translation contributes to action prepotency and whether a reduction of S-R translation ease helps to reduce the frequency of inhibitory failures.

Previous studies on unwarranted response co-execution have exclusively used target stimuli with an inherent association with the required response direction. For example, a salient onset of peripheral (left/right) visual stimuli (e.g., Kürten et al., 2022) or centrally presented directional words indicated that a left/right response should be executed (Raettig & Huestegge, 2018, 2021, 2023). In such a setting, the spatial code is available almost instantly, allowing for a quick activation of an associated, but unwarranted effector system before inhibition is complete. However, it appears plausible to assume that the ease of S-R translation should differ between, for example, peripheral and central (symbolic) stimuli (Luo & Proctor, 2018; Müller & Rabbitt, 1989). This in turn might affect the degree of action prepotency, and thereby the occurrence of inhibitory failures underlying dual-action benefits. In this case, the propensity toward inhibition failures might decrease if S-R translation ease is reduced because of a generally lower action prepotency. Furthermore, one might speculate that inhibitory failures are to a large extent instances of relatively short-latency reflexive actions (such as express saccades, e.g., Findlay & Walker, 1999; Fischer & Ramsperger, 1984; Huestegge et al., 2019) caused by peripheral stimuli instead of more “top-down controlled” actions (e.g., in response to a symbolic central stimulus). Alternatively, action prepotency might depend on dimensional (here: spatial) overlap between the imperative stimulus and the (to-be-inhibited) response. If so, the occurrence of inhibitory failures might completely vanish if there was no spatial information inherent in the stimulus at all (Kornblum et al., 1990). However, these assumptions have not yet been explicitly tested within a single experimental setup.

The present study therefore aimed to determine the impact of S-R translation ease on action prepotency and inhibitory failures in a situation requiring frequent simultaneous action execution and inhibition. To this end, we had participants switch between executing spatial (left vs. right) single manual key presses, single saccades, and manual-oculomotor dual actions in response to a single visual stimulus. Overall, we expected to replicate the strong preponderance of false-positive saccade executions in single manual trials over other types of errors (i.e., false-direction errors, false-negative errors), particularly in comparison to dual-action trials (i.e., we expected to find relative dual-action benefits). Note that false-positive key presses have been observed in such a combination of effector systems (Kürten et al., 2023), but to a much smaller degree than false-positive saccades, which is why we base our interpretations mainly on effects in the latter response modality. Critically, we also manipulated the stimulus mode and thus the ease of S-R translation^1^. A classic way of manipulating S-R translation ease is the introduction of spatial S-R incompatibility: For example, it is more difficult to execute a “right” response based on a stimulus indicating a “left” response (Fitts & Seeger, 1953; Kornblum et al., 1990). However, in the current setting, such a manipulation of S-R translation ease would introduce a spatial conflict between the prepotent but unwarranted response tendency and the actually required response in single-action trials. For instance, in an incompatible single-manual trial a stimulus indicating a “left” response would create a general tendency towards a left response that would have to be overcome only for the manual response but not for the unwarranted oculomotor response. To avoid such a potential confound and to allow for a better comparison with previous SDS studies, we therefore resorted to a procedure typically used in cueing paradigms, namely the utilization of peripheral versus central (symbolic) stimuli (Posner, 1980, 2016; Remington, 1980). Specifically, we used three types of visual stimuli: (1) a salient peripheral (left vs. right) square, (2) a centrally presented arrow pointing to the left or to the right, and (3) a centrally presented shape arbitrarily mapped to left versus right responses. We reasoned that the onset of the peripheral square should be translated into the corresponding spatial response code in a most direct, quasi-automatic manner due to its inherent spatial properties (Lu & Proctor, 1995; Müller & Rabbitt, 1989). With the central arrow being a symbolic stimulus, participants must resort to learned (symbolic) conventions to extract its meaning (Miles & Proctor, 2012). As a consequence (and similar to exogeneous vs. endogenous cues utilized in typical cueing paradigms, see Posner, 1980, 2016), arrows should imply a somewhat reduced S-R translation ease when compared with the peripheral stimuli. Finally, participants should have the strongest difficulties with translating the arbitrary central shape into left (vs. right) response codes due to the lack of any (physical or symbolic) dimensional overlap (Kornblum et al., 1990). On a neurophysiological level, this is reflected, for example, by later lateralized readiness potentials with arbitrary central (color) cues compared to central arrow cues signaling eventual response direction (Eimer, 1995; Eimer et al., 1995). The three levels of S-R translation ease should be reflected by increasing corresponding RT levels in either response modality (RT: peripheral square < central arrow < central shape), which thus serves as a manipulation check. Central to the present study, however, was the effect of the S-R translation ease manipulation on error rates, particularly, on false-positive saccade executions in single manual trials as indications of inhibitory failures.

The following prediction alternatives were made based on competing theoretical assumptions. First, the frequency of inhibitory failures could decrease with decreasing S-R translation ease: Activation from the spatial stimulus should spread more quickly to an unwarranted saccade code the faster this spatial information is available, granting less time for inhibitory control. Specifically, if inhibition of the saccade in single-manual trials proceeds in parallel with (and independent from) S-R translation as is usually assumed in independent race models of inhibitory control (Logan & Cowan, 1984; Matzke et al., 2018), any prolongation of this process should yield better inhibitory control (*extended time for inhibitory control* account). Taken together, this reasoning would be reflected by a *decreasing* rate of false-positive saccades with increasing RTs (due to more difficult S-R translation). Second, an *increasing* rate of false-positive saccades with increasing RTs (due to more difficult S-R translation) could also be expected, for example, based on the assumption of fewer processing resources being available for effector system inhibition due to a more difficult direction specification. This would, of course, require a dependence of effector system inhibition and S-R translation on a common, limited processing capacity (*translation-inhibition conflict* account, see e.g., Verbruggen & Logan, 2015). Finally, a third possible outcome would be a comparable frequency of false-positive saccades across levels of S-R translation ease. This would, on a theoretical level, indicate that the failure (or success) of effector system inhibition was (at least in part) determined independently from (i.e., before) the specification of the spatial response parameters conveyed by the imperative stimulus (*hierarchical action specification* account). This prediction appears reasonable based on studies in the domain of manual motor control demonstrating that the selection of the hand (effector) occurs prior to target specification in manual aiming movements (Herbort & Rosenbaum, 2014).

Any potential differences in inhibitory failure rates across conditions could be further affected by two additional mechanisms. First, false-positive errors could largely represent short-latency reflexive actions such as express saccades (Findlay & Walker, 1999; Fischer & Ramsperger, 1984; Meeter et al., 2010) executed independently from the actually required (manual) action. If this is the case, we would expect particularly frequent false-positive saccades with the peripheral square stimulus (*reflexive inhibition failures*). If, in contrast, false-positive errors represent failures to implement the proper behavior during action selection (i.e., when saccades are co-activated when the time has come to initiate the manual response as posited by the spreading activation model described above), we would not expect exceptionally high rates of false-positive saccades with the peripheral stimulus (Everling & Johnston, 2013). Finally, if spreading activation leading up to false-positive errors was mainly triggered by the stimulus, for example, due to spatial dimensional overlap with the to-be-inhibited response, we should observe false-positive saccades and a potential modulation by S-R translation ease with the peripheral square and the central arrow but not with the central shape stimulus (*stimulus-based co-activation*, see Kornblum et al., 1990). If inhibitory failures were instead triggered not only by strong a-priori stimulus-response associations but by a strong coupling between both effector systems, we should instead observe frequent false-positive saccades even with the central square stimulus (*action-based co-activation*).

## Methods

Preregistration, raw data, and analysis scripts are available at https://osf.io/9shkf/?view_only=9d93819f46b746449803eabbd579036a.

### Participants

Based on a previous study (Kürten et al., 2023), we determined that a sample size of 10 would suffice to demonstrate accuracy-related dual-action benefits in saccade errors (main effect action demand, ${\widehat{\eta }}_{p}^{2}$ = 0.544) with a power of 1-$\beta$ = 0.95 at a significance level of $\alpha$ = 0.05. To achieve sufficient power to find a potential modulation of dual-action benefits by stimulus mode, we decided to collect data from forty-eight volunteers (mean age = 25.1 years, SD = 4.4, 76.79% female, 98.21% right-handed) with normal or corrected-to-normal vision (without color blindness). Eight data sets were replaced with the help of new participants due to either missing data (2 data sets), a global error rate of > 30% (6 data sets), and/or due to producing < 10 valid correct RTs in any of the experimental conditions (3 data sets). All participants gave their informed consent and received monetary compensation.

### Apparatus, stimuli and procedure

**Fig. 1:**
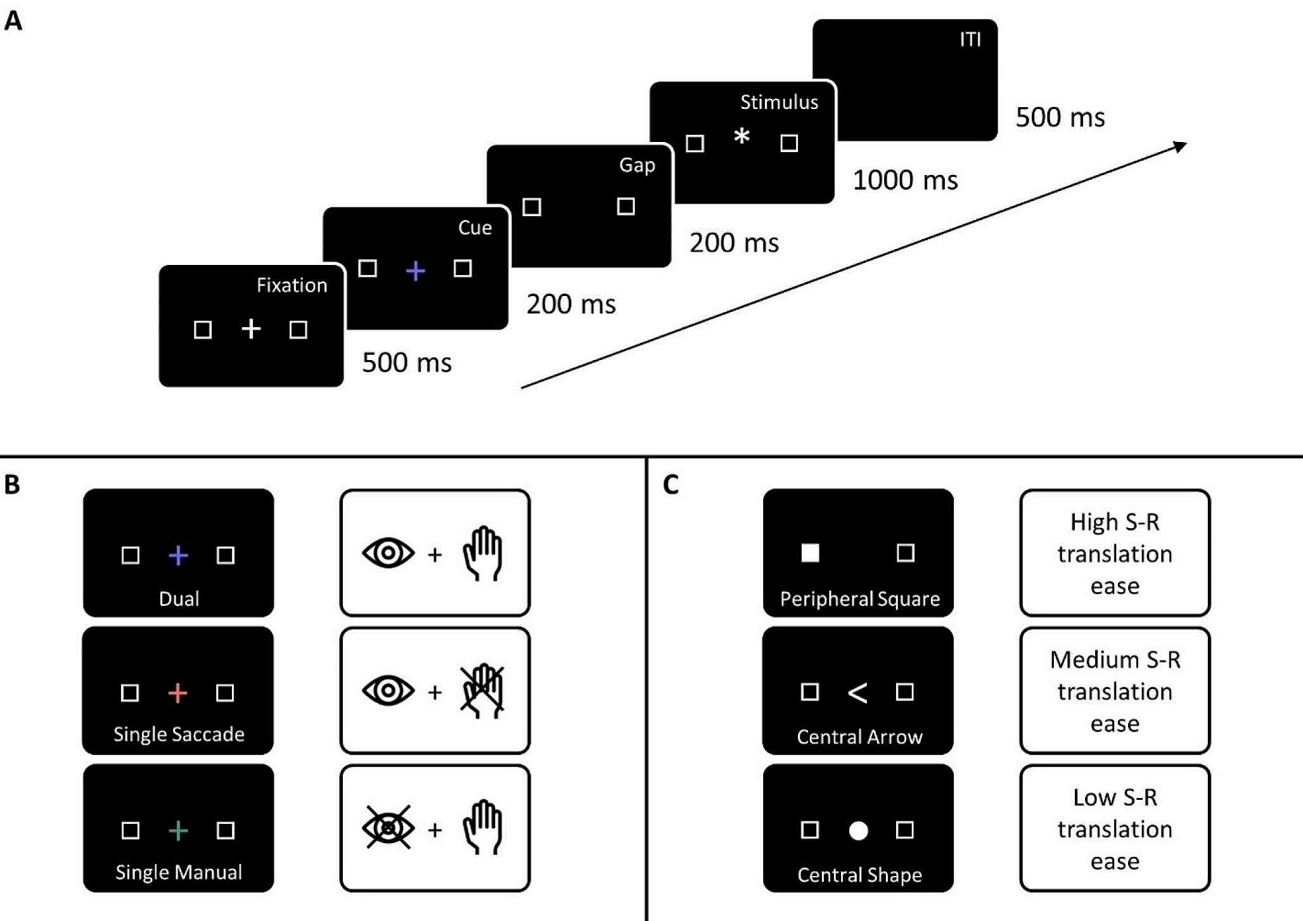
Experimental setup. **A**. Schematic trial structure. **B**. Exemplary color-to-action demand mappings (counterbalanced across participants). **C**. Stimulus modes. For each stimulus mode, 24 practice trials followed by three experimental blocks of 60 trials each (i.e., 20 per action demand, 10 per action demand and direction) were administered. See the online article for the color version of this figure

After giving their informed consent, participants were seated in front of a 21” cathode-ray tube monitor with a spatial resolution of 1024 × 768 pixels and a temporal resolution of 100 Hz at a viewing distance of 67 cm. Latencies and directions of eye movements of the right eye were recorded using an EyeLink 1000 eye-tracking system (SR Research, Ontario, Canada) with a sampling rate of 1000 Hz. Head movements were minimized using a chinrest with forehead support. Manual responses of the left and right index fingers were recorded using two buttons on a Cedrus response pad (Cedrus corporation) placed on the desk instead of the regular computer keyboard (and thus not in the line of sight of the participants). All stimuli were presented against a black background. The experiment comprised three sub-sessions, one for each stimulus mode, consisting of written and verbal instructions followed by a 24-trial training block that could be repeated in case of insufficient task understanding and three experimental blocks with 60 trials each (20 per action demand, 10 per action demand and direction). The order of stimulus-mode sub-sessions was counterbalanced across participants. Figure 1 depicts a schematic trial structure. Each trial started with the presentation of the central fixation cross at the screen center (a plus sign, size 10 pixels, 0.33°VA) and two saccade landing targets (black squares with white borders size 10 pixels, 0.33°VA, border width 1 pixel) at an eccentricity of 8°VA to the left and right of the screen center. After 500 ms, the cross changed color to either red, green or blue to indicate action demand (e.g., red = single saccade, green = single manual, blue = dual, mapping counterbalanced across participants). After 200 ms the colored cross disappeared while the saccade targets remained visible. After another 200 ms, the imperative stimulus was presented for 1000 ms. If no response was recorded within this time window, an error message (“Zu langsam”, German for “Too slow”) was displayed in white Times New Roman font (size 20) for 1000 ms. No other error feedback was given. Three types of stimuli were used. (1) A white square (size 10 pixels, 0.33°VA) that filled either the left or the right saccade landing target, (2) a white arrow (size 10 pixels, 0.33°VA) presented at the screen center, and (3) a white shape (diamond and circle, size 10 pixels, 0.33°VA) presented at the screen center. Trials were separated by a 500 ms blank-screen inter-trial interval (ITI). Participants responded spatially (left vs. right) with a manual button press, with a saccadic eye movement to one of the (left/right) saccade landing targets, or with both responses. Responses to the peripheral stimulus and central arrow were always spatially compatible to the stimulus (i.e., left peripheral square/left-pointing central arrow required a left button press and/or a leftward saccade and vice versa). The mapping of the central shape to response direction was arbitrary (e.g., diamond = left, circle = right) and counterbalanced across participants. The experiment was programmed using Experiment Builder software (version 2.3.527, SR Research).

### Data analysis

Reaction times (RTs, ms) and error rates (ERs, %) served as dependent variables. Practice blocks were removed from all analyses. From the remaining nine experimental blocks, we removed premature responses (RTs < 50 ms, 0.34% of all trials). The remaining trials were analyzed for errors, including false-direction errors (i.e., wrong button pressed/saccade toward the wrong target), false-negative errors (e.g., no saccade executed in single saccade/dual-action trials) and, indicating inhibition failure, false-positive errors (e.g., saccade executed in single manual trials). From RT analyses, we removed all error trials (14.14% of all trials) and outliers (RT > 3 SDs away from cell mean, 1.84% of all trials). In total, 15.91% of all trials were removed from RT analyses. RTs and ERs were subjected to repeated measures analyses of variance (ANOVAs) with the within-subject independent variables action demand (single manual, single saccade, dual) and stimulus mode (peripheral, central arrow, central shape). Since errors in both response modalities could occur in every trial, saccades and manual responses were analyzed separately. Note that ERs comprised different types of errors that can occur at different action demands in each response modality (see Table 1). For example, false-positive saccade errors can only occur in single manual trials while false-positive manual errors can only occur in single saccade trials. In RT analyses, the factor action demand was effectively reduced to two levels (single vs. dual) for each response modality since we only included correct responses (i.e., no saccade RTs in single manual trials, no manual RTs in single saccade trials). The significance level was set to $\alpha$ = 0.05 for main analyses. Greenhouse-Geisser corrections were routinely applied to within-subject factors with more than two levels. Significant main effects of factors with more than two levels and significant interaction effects were followed up by Bonferroni-corrected pairwise comparisons. Data processing and analysis was conducted in R (R core team), the papaja package (Aust & Barth, 2014/2022). was used to write a computationally reproducible methods and results section. Additionally, we employed the TOSTER R package (Lakens & Caldwell, 2023) for equivalence tests on false-positive ERs, specifically saccade errors in single-manual trials and manual errors in single-saccade trials. This procedure was aimed at assessing potential modulations by stimulus mode within the context of a previously observed reduction of false-positive ERs by extending preparation time between cue and imperative stimulus from 100 ms to 400 ms (Kürten et al., 2023). Equivalence bounds were set to 12% for false-positive saccades and to 2% for false-positive manual key presses. Equivalence tests were corrected for multiple comparisons based on a suggestion by Lauzon and Caffo (2009).

**Table 1:** Possible error types for different action demands and response modalities

Action Demand	Saccade Modality	Manual Modality
Dual	false-direction & false-negative	false-direction & false-negative
Single Saccade	false-direction & false-negative	false-positive & false-direction
Single Manual	false-positive & false-direction	false-direction & false-negative

Note. Saccades in single manual trials and key presses in single saccade trials are always false-positive errors, regardless of whether executed in the correct direction or not

## Results

### Correct RT data

**Fig. 2:**
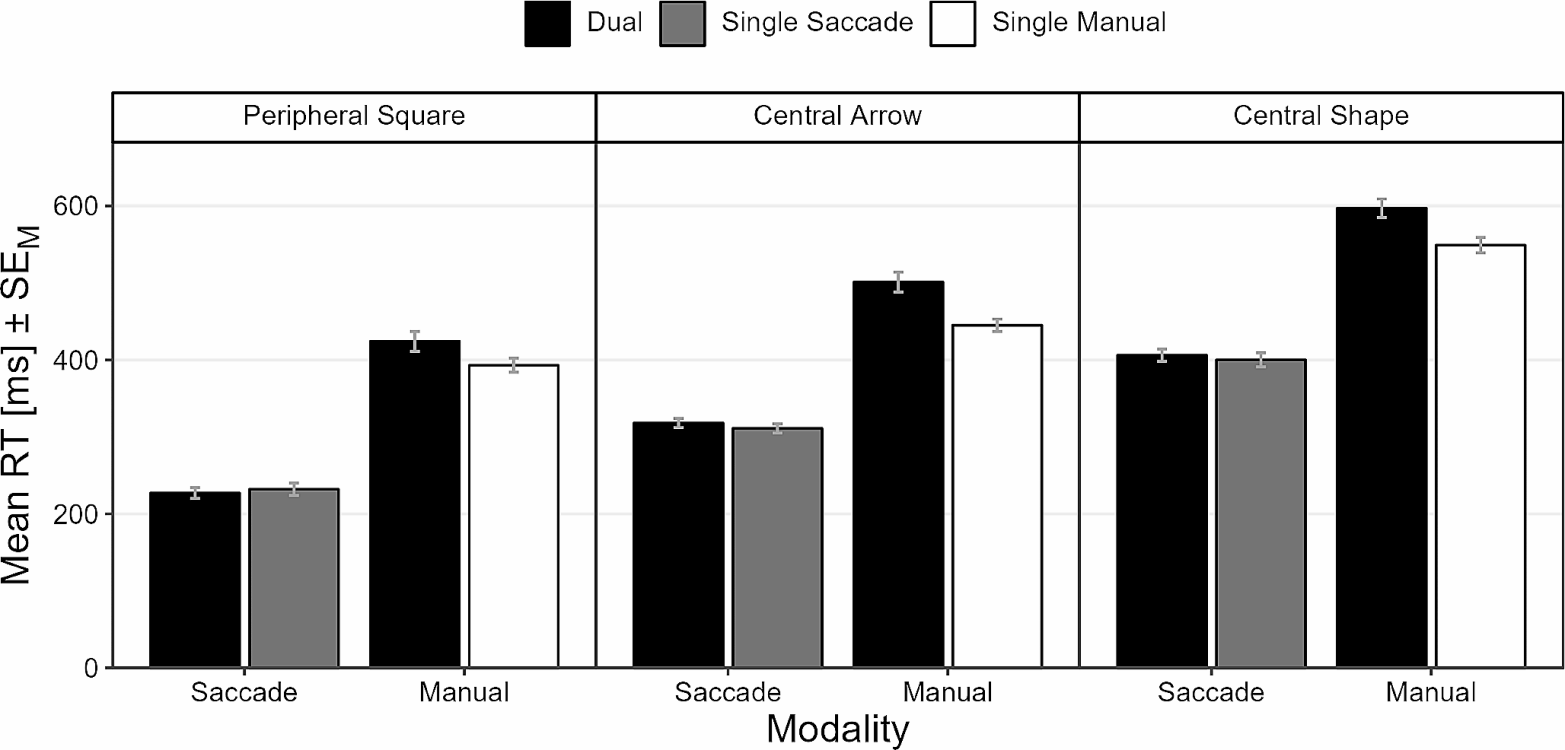
Correct reaction time data. Correct RTs (ms) as a function of action demand (dual, single saccade, single manual) and stimulus mode (peripheral square, central arrow, central shape). Every saccade (manual) response in single manual (single saccade) trials represented a false-positive error and thus produced no correct reaction time. Error bars represent standard error of the mean (SE_M_)

Complete descriptive summary statistics can be obtained from Table S1 in the supplementary material. Correct RTs of both response modalities as a function of action demand (trial type) and stimulus mode are displayed in Fig. 2.

*Saccade RTs*. The main effect of action demand was not significant, $F(1,47)=0.09$, $p=0.766$, ${\widehat{\eta }}_{p}^{2}=0.002$. The main effect of stimulus mode was significant, $F(1.61,75.53)=316.90$, $p<0.001$, ${\widehat{\eta }}_{p}^{2}=0.871$. Saccade responses to the peripheral square (*M* = 229 ms) were faster than saccade responses to the other two stimulus modes (both *p*s < 0.001). Saccade responses to the central arrow (*M* = 314 ms) were faster than saccade responses to the central shape (*M* = 402 ms, *p* < 0.001). The interaction of action demand and stimulus mode was not significant, $F(1.84,86.50)=2.37$, $p=0.104$, ${\widehat{\eta }}_{p}^{2}=0.048$.

*Manual RTs*. The main effect of action demand was significant, $F(1,47)=41.71$, $p<0.001$, ${\widehat{\eta }}_{p}^{2}=0.470$. Manual responses were, overall, faster in single manual trials (*M* = 462 ms) than in dual-action trials (*M* = 505 ms). The main effect of stimulus mode was significant as well, $F(1.62,76.00)=204.71$, $p<0.001$, ${\widehat{\eta }}_{p}^{2}=0.813$. Manual responses to the peripheral square (*M* = 408 ms) were faster than manual responses to the other two stimulus modes (both *p*s < 0.001). Manual responses to the central arrow (*M* = 472 ms) were faster than manual responses to the central shape (*M* = 571 ms, *p* < 0.001). The interaction of action demand and stimulus mode was significant, $F(1.98,93.29)=4.60$, $p=0.013$, ${\widehat{\eta }}_{p}^{2}=0.089$. Dual-action costs were significant for all stimulus modes (all *p*s < 0.001) but they were largest for the central arrow stimulus ($\varDelta M=55$ms).

### Error data

**Fig. 3:**
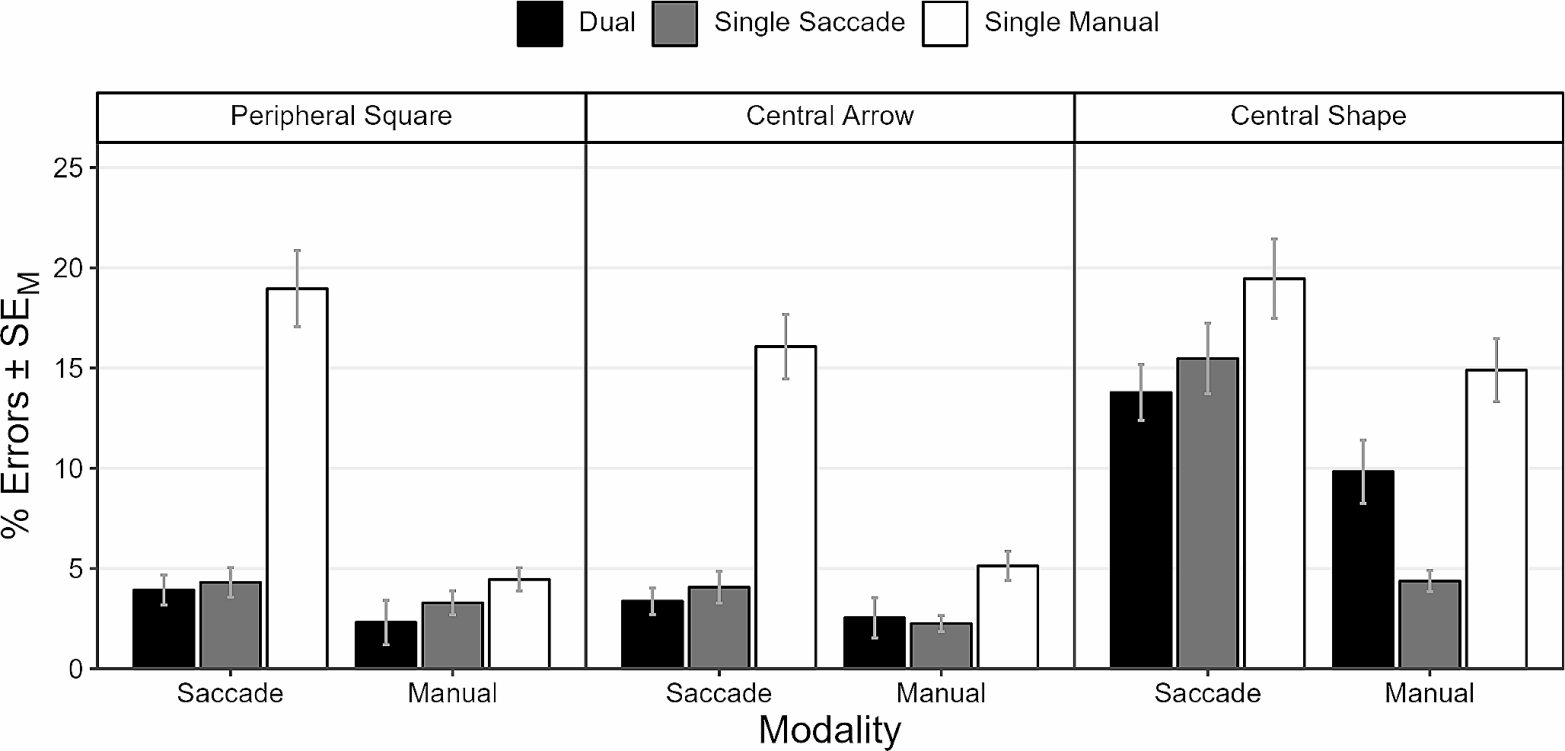
Error rates as a function of stimulus mode and action demand. Note. ERs (%) of both response modalities (saccade, manual) as a function of action demand (dual, single saccade, single manual) and stimulus mode (peripheral square, central arrow, central shape). Error bars represent the standard error of the mean (SE_M_)

**Fig. 4:**
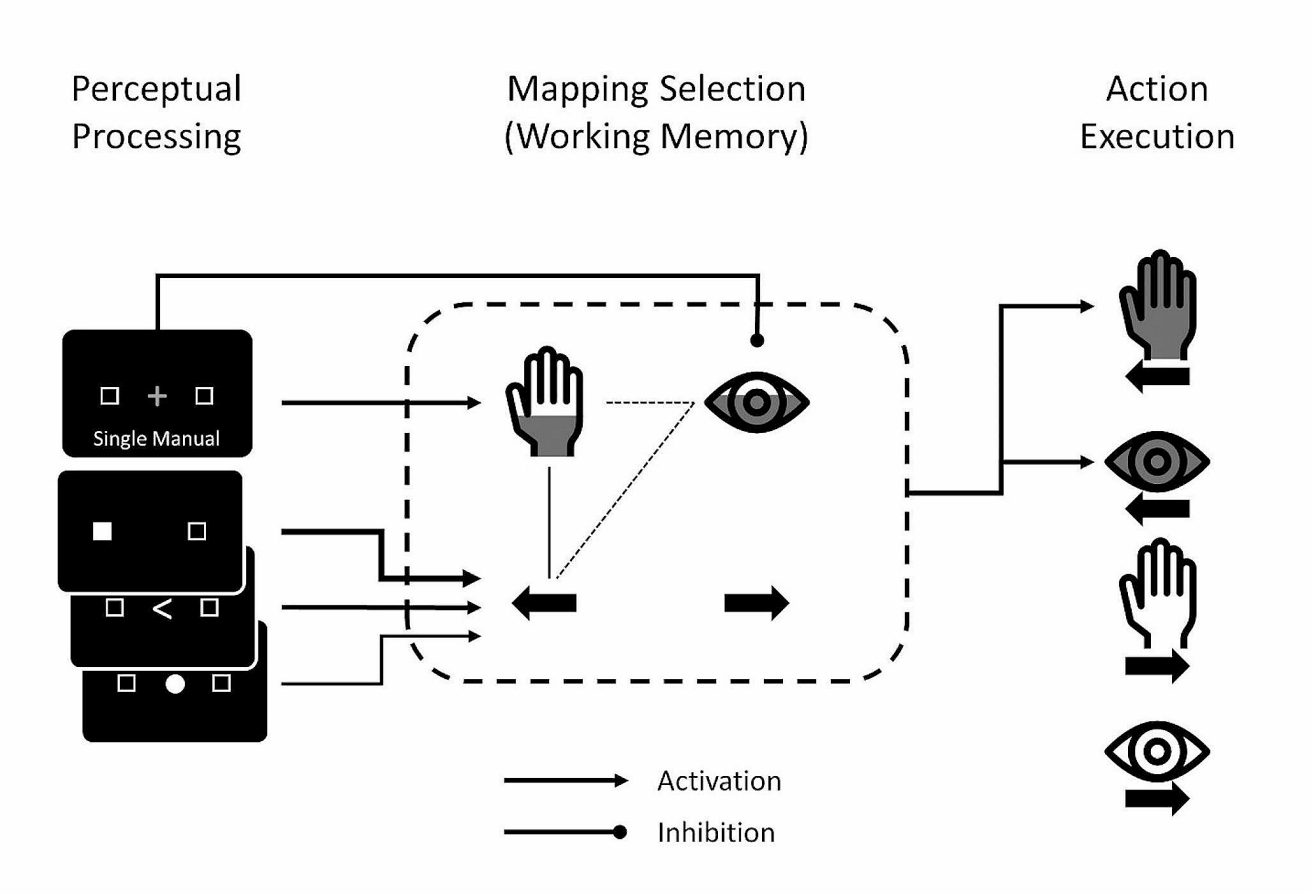
Conceptual framework of (false-positive) action selection. A framework of action selection in single manual trials for the three different stimulus modes. The perceptual processing stage contains cue and imperative stimulus. The mapping selection stage (dashed box) contains potentially action-relevant modality codes (hand symbol = manual system, eye symbol = oculomotor system) and spatial codes (left-pointing arrow = “left”, right-pointing arrow = “right”) in working memory. The “fill level” of the modality codes indicate action prepotency (oculomotor system > manual system). Solid lines without arrowheads represent the currently relevant binding between the manual modality code and the “left” spatial code, dashed lines without arrow heads represent erroneously spreading activation due to frequent association. The action execution stage contains all potential task-relevant actions (left keypress, leftward saccade, right keypress, rightward saccade). Filled symbols designate the actually executed actions within a trial. In a single-manual trial, the colored cue activates the manual modality code (and prompts inhibition of the oculomotor modality), the stimuli activate the “left” spatial code at a different rate depending on S-R translation automaticity (indicated by line strength from stimulus to spatial code). Activation spreading from the manual and the “left” code to the prepotent saccade code (action-based co-activation of the other effector system) that counteracts the original inhibitory command based on the cue eventually resulting in execution of a false-positive leftward saccade along with the required left keypress. See the online article for the color version of this figure.

Figure 3 displays ERs as a function of action demand and stimulus mode. *Saccade ERs*. The main effect of action demand was significant, $F(1.17,54.97)=72.23$, $p<0.001$, ${\widehat{\eta }}_{p}^{2}=0.606$. Saccade errors were least frequent in dual-action trials (*M* = 7.03%), not significantly more frequent in single saccade trials (*M* = 7.96%, *p* = 0.100) and, crucially, most frequent in single manual trials (*M* = 18.16%, both *p*s < 0.001) indicating overall dual-action benefits due to frequent false-positive saccades. The main effect of stimulus mode was significant as well, $F(1.53,72.10)=30.28$, $p<0.001$, ${\widehat{\eta }}_{p}^{2}=0.392$. Saccade errors were least frequent with the central arrow stimulus (*M* = 7.84%) but were not significantly less frequent compared to the peripheral square stimulus (*M* = 9.07%, *p* = 0.401). Saccade errors were most frequent with the central shape stimulus (*M* = 16.24%, both *p*s < 0.001). The interaction of action demand and stimulus mode was significant, $F(2.75,129.16)=14.17$, $p<0.001$, ${\widehat{\eta }}_{p}^{2}=0.232$. Visual inspection of Fig. 3 suggests that this interaction was mainly driven by the high ERs in dual-action and single saccade trials in conditions involving the central shape stimulus and Fig. 4 indicates that the these mainly contained false-direction errors instead of false-negative errors indicating difficulties with correctly classifying the arbitrary central shape. Saccade errors in single manual trials (false-positive saccades) were significantly more frequent than saccade errors in dual-action trials (false-direction & false-negative saccade errors) with all stimulus modes (all *p*s < 0.003). Crucially, however, false-positive saccade ERs were on a high level across all stimulus modes. Bonferroni-corrected comparisons of false-positive saccade ERs in single manual trials yielded no significant differences (all *p*s > 0.066). However, based on exploratory equivalence tests, we could establish equivalence between stimulus modes regarding false-positive errors within equivalence bounds of ± 12% points (all ps < 0.001).

*Manual ERs*. The main effect of action demand was significant, $F(1.77,83.30)=10.61$, $p<0.001$, ${\widehat{\eta }}_{p}^{2}=0.184$. Manual ERs did not differ significantly between dual-action trials (*M* = 4.89%) and single saccade trials (*M* = 3.31%, *p* = 0.502). Manual errors were, more frequent in single manual trials (*M* = 8.16% compared to the other two action demands (both *p*s < 0.027). The main effect of stimulus mode was significant as well, $F(1.25,58.58)=58.60$, $p<0.001$, ${\widehat{\eta }}_{p}^{2}=0.555$. The frequency of manual errors with the peripheral square stimulus (*M* = 3.35%) did not differ significantly from those with the central arrow stimulus (*M* = 3.31%, *p* > 0.999). Manual errors were more frequent with the central shape stimulus (*M* = 9.70%) compared to the other two stimulus modes (both *p*s < 0.001). Finally, the interaction of action demand and stimulus mode was significant, $F(2.74,128.90)=21.45$, $p<0.001$, ${\widehat{\eta }}_{p}^{2}=0.313$. This was mainly due to the high manual ERs in dual-action trials and single saccade trials in conditions involving the central shape stimulus. False-positive manual ERs in single saccade trials did not differ significantly between conditions involving the peripheral and the central arrow stimuli nor between conditions involving the peripheral and the central shape stimuli (both *p*s > 0.134), but they did differ between conditions involving the central arrow and the central shape stimuli (*p* < 0.001). Based on exploratory equivalence tests, we could not reject the equivalence of false-positive manual ERs across stimulus modes within bounds of ± 2% points (all *p*s > 0.062).

## Discussion

The present study investigated the effect of S-R translation ease on inhibitory failures in a setting that sometimes required the execution of one (e.g., manual) response and the simultaneous inhibition of another (e.g., saccade response). The main results can be summarized as follows. Correct RTs in both response modalities steadily increased as a function of stimulus mode (RT: peripheral square < central arrow < central shape), indicating that the manipulation of S-R translation ease indeed worked as intended. While saccades showed a comparable latency in single-action and dual-action trials, we observed dual-action costs for the manual key press across stimulus modes. This could indicate a motor bottleneck, since the key press was usually executed after the initiation and completion of the saccade (Bratzke et al., 2008, 2009) and/or prioritization of the “saccade part” of the oculomotor-manual dual action (Hoffmann et al., 2019; Pieczykolan & Huestegge, 2014). Qualitative differences between the two inherently spatial stimulus modes (peripheral squares and central arrows) and the non-spatial stimulus mode (central shapes) are reflected by a substantially higher frequency of directional and false-negative errors in the latter mode compared to the former modes (for a depiction of error rate as a function of stimulus mode and error type, see Figure S1 in the supplemental material). Thus, as expected, the presence of spatial information within the stimulus substantially improved the ability to select the appropriate response direction. Again, this corroborates the effectiveness of our manipulation of S-R translation ease. The main finding of the present study, however, was the relative independence of the frequency of false-positive saccades in single-manual trials from the manipulation of S-R translation ease.^2^

### Stimulus-based and action-based co-activation

False-positive saccades with very low (< 100 ms) RTs occurred more often with the peripheral square stimulus (3.46% of false-positive saccades) compared to the central arrow stimulus (0.86%) and the central shape stimulus (0.53%). However, false-positive saccades were clearly not limited to such express saccades, ruling out a simple *reflexive inhibitory failures*. This is remarkable given that we employed a blank screen gap between the offset of the central cue and the onset of the (peripheral or central) stimulus, a setting that has been shown to elicit a rather large amount of express saccades in situations without additional manual response demands (e.g., Fischer & Ramsperger, 1984). Further, erroneous saccade co-execution was not dependent on inherently spatial stimuli as there was no sharp drop in false-positive saccade rates in the central shape stimulus condition compared to the other conditions, speaking against a purely *stimulus-based co-activation* mechanism. Instead, it seems that false-positive saccade generation was tightly linked to the concurrent processing of the manual response, *indicating action-based co-activation* as an additional mechanism. Most importantly, even though RT levels within the different stimulus modes suggested a clear hierarchy of S-R translation ease, we did not find a corresponding gradual modulation of false-positive saccade frequency. We even found evidence for significant statistical equivalence regarding the false-positive ERs. The results thus quite clearly demonstrate that the overall frequency of false-positive saccades was rather unaffected by stimulus mode. Thus, we found no support for an *extended time for inhibitory control* account. It seems unlikely that participants simply used the extra time provided by prolonged S-R translation processes for (a) completing the selection of the proper effector system and (b) inhibiting the unwarranted effector system after the imperative stimulus was presented (as false-positive saccade frequency did not gradually decrease with S-R translation ease). On the other hand, the frequency of false-positive saccades also did not gradually increase as S-R translation ease decreased as predicted by a *translation-inhibition-conflict* account. Inhibitory control did not seem to suffer substantially from any limited capacity being diverted to S-R translation demands. This indicates that success (or failure) of saccade inhibition was, to some extent, already determined before the direction of the action was specified, compatible with a *hierarchical action specification* account.

This particular assumption is consistent with a model of saccade programming differentiating between a WHEN and WHERE pathway that are processed rather independently and potentially sequentially in our particular paradigm (Findlay & Walker, 1999; Huestegge et al., 2019). In addition, it is also compatible with more general motor programming studies (Rosenbaum, 1985) demonstrating that some action parameters (e.g., the hand used for aiming) are selected independently of other parameters (e.g., the target to aim for, see Herbort & Rosenbaum, 2014). Conversely, inhibitory control to prevent a false-positive saccade would need to act at the level of the effector system per se rather than on an already spatially specified saccade (Kürten et al., 2023).

Generation of a false-positive saccade in single-manual trials can be readily explained within the model of action selection proposed by Huestegge and Koch (2010, see also Huestegge, 2011). An illustration of the proposed mechanisms is depicted in Fig. 4. Proactive inhibition of the prepotent saccade system is initiated by the prior cue (Greenhouse et al., 2012). This is counteracted by activation spreading to the saccade modality code both from the spatial code (triggered by the stimulus, stimulus-based co-activation) as well as from the frequently associated manual modality code (action-based co-activation). Activating the saccade code above a certain threshold results in the false-positive execution along with the required manual response. False-positive saccades are likely preceded by competition between executory and inhibitory processes, given that they tend to have, on average, longer latencies, and smaller amplitudes than correct saccades (see Table S2 and Figure S2 in the supplementary material). This ties in with studies demonstrating a negative impact of increasing mental workload on saccadic latency and amplitude (e.g., Huestegge & Koch, 2009; May et al., 1990; Spering, 2022; Stuyven et al., 2000). It is important to note that the present framework is functional in nature rather than physiological. However, detailed physiologically grounded models of various processes relevant to the current results exist. Particularly, there is a large body of research on saccade initiation and inhibition in the stop-signal and anti-saccade paradigms mediated by fixation neurons in the superior colliculus (e.g., Bompas et al., 2020; Coe & Munoz, 2017). Another field potentially relevant to the current results is eye-hand coordination in reaching/pointing toward common targets controlled by a parieto-frontal network (e.g., Battaglia-Mayer et al., 2006; Mascaro et al., 2003). It should be noted, however, that these models might not directly apply to the current situation given the differences in the paradigms used. While exceeding the scope of the current study, future work may thus be dedicated to generalizing these models to the current setting yielding erroneous saccade co-execution.

### Saccade prepotency versus dual-action prepotency

Of course, most single-manual trials with a false-positive saccade also involved the execution of the required manual response.^3^ The majority of false-positive saccades went into the same direction as the required manual response (see Figure S3 in the supplemental material). An interesting issue is therefore how these “erroneous dual actions” compare to the “correct dual actions” initiated in dual-action trials, as this has repercussions on what behavior actually is *prepotent* in single-manual trials: executing a saccade, or executing an oculomotor-manual dual action. On the one hand, false-positive saccades might be initiated rather independently from the required manual response. This would be in line with the idea that the saccade per se is prepotent. On the other hand, false-positive saccades could be part of a “Gestalt-like” compound action that is erroneously executed in some of the single-manual trials (of course, a mixture of both could also be assumed). It is very difficult to finally decide between these alternatives based on the current data. However, a preliminary answer could be generated by comparing the correlations of saccade and manual RTs and the inter-response intervals (IRIs) between correct and erroneous dual actions. Specifically, correlations between saccade and manual RTs were, on average, much lower in erroneous dual actions compared to correct dual actions (see Table S3 in the supplementary material). Additionally, IRIs in *correct* dual actions were distributed in a unimodal manner with a rather small variability indicative of compound responding (cf. Huestegge & Koch, 2009). In contrast, IRIs of erroneous dual actions were much more variable (see Figure S4 in the supplementary material). These exploratory analyses should be treated with caution, of course, given the low and variable (across participants) number of single-manual trials involving an erroneous dual action. However, they tentatively indicate that the saccade per se (and not an oculomotor-manual compound action) was prepotent in single-manual trials. This prepotent saccade was then erroneously triggered by the requirement to produce a frequently associated manual response. Such a mechanism of erroneous co-activation of a currently unwarranted response modality is reminiscent of the concept of response-response priming (Moeller & Frings, 2019a, b; see also Hommel, 2020). In corresponding experiments, it has been shown that the repetition (vs. change) of a response can retrieve (and thus facilitate) a recently associated response (independent from a stimulus repetition or change), even across different effector systems (Moeller & Frings, 2019c).

## Conclusion

To conclude, the results of the current study allow us to further specify the mechanisms underlying erroneous (saccade) co-execution in situations requiring only a single (manual) response. Generally, the results emphasize the robustness of dual-action benefits characterized by false-positive responses in single-action contexts. Dual actions were, overall, easier to execute than single (manual) actions due to costly inhibitory demands for the latter. Importantly, the frequency of false-positive saccades in single-manual trials did not meaningfully depend on the ease of the oculomotor S-R translation process, ruling out simple explanations in terms of *extended time for inhibitory control, translation-inhibition conflicts*, or *reflexive false-positive (express) saccades*. Instead, they appeared to be tightly linked to the execution of a secondary manual response (*action-based co-execution*) and to be determined largely at the level of effector system representations, not at the level of a fully specified response (in line with a *hierarchical action specification account*).

The authors have no competing interests to declare that are relevant to the content of this article.

## Electronic supplementary material

Below is the link to the electronic supplementary material.

Supplementary Material 1

## Footnotes

1 Note that in the SDS paradigm, which involves a cue indicating response type (e.g., single manual, single saccade, dual) and a separate target stimulus indicating response direction, a full specification of the required response(s) can only be achieved by combining cue and target stimulus information. To stay within common terminology, here and in the following we refer to stimulus-*response* translation ease, although, strictly speaking, we manipulated the ease of stimulus-response *direction* translation.

2 We observed false-positive manual responses in single-saccade trials, indicating that even the latter trial type incurred some level of inhibitory demand. However, due to the generally low frequency of false-positive manual errors (< 5%), our interpretations are based solely on the data from the saccade modality.

3 Around 20% of single-manual trials containing a false-positive saccades did not contain the required manual response (i.e., false-positive saccade error *and* false-negative manual error). We assume that this was mainly caused by the slower manual action not being recorded within the relatively short response window of 1000 ms, not by the selection of only a single saccade.

## References

[CR1] Aust, F., & Barth, M. (2022). *papaja: Prepare reproducible APA journal articles with R Markdown* (Version 0.2.0) [Computer software]. https://github.com/crsh/papaja (Original work published 2014).

[CR2] Battaglia-Mayer, A., Archambault, P. S., & Caminiti, R. (2006). The cortical network for eye-hand coordination and its relevance to understanding motor disorders of parietal patients. *Neuropsychologia*, *44*(13), 2607–2620. 10.1016/j.neuropsychologia.2005.11.021. 16458334 10.1016/j.neuropsychologia.2005.11.021

[CR3] Bompas, A., Campbell, A. E., & Sumner, P. (2020). Cognitive control and automatic interference in mind and brain: A unified model of saccadic inhibition and countermanding. *Psychological Review*, *127*(4), 524–561. 10.1037/rev0000181. 31999149 10.1037/rev0000181 PMC7315827

[CR5] Bratzke, D., Ulrich, R., Rolke, B., Schröter, H., Jentzsch, I., & Leuthold, H. (2008). Motor limitation in dual-task processing with different effectors. *Quarterly Journal of Experimental Psychology (2006)*, *61*(9), 1385–1399. 10.1080/17470210701536856. 19086191 10.1080/17470210701536856

[CR4] Bratzke, D., Rolke, B., & Ulrich, R. (2009). The source of execution-related dual-task interference: Motor bottleneck or response monitoring? *Journal of Experimental Psychology: Human Perception and Performance*, *35*(5), 1413–1426. 10.1037/a0015874. 19803646 10.1037/a0015874

[CR6] Coe, B. C., & Munoz, D. P. (2017). Mechanisms of saccade suppression revealed in the anti-saccade task. *Philosophical Transactions of the Royal Society B: Biological Sciences*, *372*(1718), 20160192. 10.1098/rstb.2016.0192. 10.1098/rstb.2016.0192 PMC5332851 28242726

[CR7] Eimer, M. (1995). Stimulus-response compatibility and automatic response activation: Evidence from psychophysiological studies. *Journal of Experimental Psychology: Human Perception and Performance*, *21*(4), 837–854. 10.1037/0096-1523.21.4.837. 7643051 10.1037//0096-1523.21.4.837

[CR8] Eimer, M., Hommel, B., & Prinz, W. (1995). S-R compatibility and response selection. *Acta Psychologica*, *90*(1), 301–313. 10.1016/0001-6918(95)00022-M.

[CR9] Everling, S., & Johnston, K. (2013). Control of the superior colliculus by the lateral prefrontal cortex. *Philosophical Transactions of the Royal Society B: Biological Sciences*, *368*(1628), 20130068. 10.1098/rstb.2013.0068. 10.1098/rstb.2013.0068 PMC3758210 24018729

[CR10] Fagot, C., & Pashler, H. (1992). Making two responses to a single object: Implications for the central attentional bottleneck. *Journal of Experimental Psychology: Human Perception and Performance*, *18*(4), 1058–1079. 10.1037/0096-1523.18.4.1058. 1431744 10.1037//0096-1523.18.4.1058

[CR11] Findlay, J. M., & Walker, R. (1999). A model of saccade generation based on parallel processing and competitive inhibition. *Behavioral and Brain Sciences*, *22*(4), 661–674. 10.1017/S0140525X99002150. 11301526 10.1017/s0140525x99002150

[CR12] Fischer, B., & Ramsperger, E. (1984). Human express saccades: Extremely short reaction times of goal directed eye movements. *Experimental Brain Research*, *57*(1), 191–195. 10.1007/BF00231145. 6519226 10.1007/BF00231145

[CR13] Fitts, P. M., & Seeger, C. M. (1953). S-R compatibility: Spatial characteristics of stimulus and response codes. *Journal of Experimental Psychology*, *46*(3), 199–210. 10.1037/h0062827. 13084867 10.1037/h0062827

[CR14] Greenhouse, I., Oldenkamp, C. L., & Aron, A. R. (2012). Stopping a response has global or nonglobal effects on the motor system depending on preparation. *Journal of Neurophysiology*, *107*(1), 384–392. 10.1152/jn.00704.2011. 22013239 10.1152/jn.00704.2011 PMC3349702

[CR15] Herbort, O., & Rosenbaum, D. A. (2014). What is chosen first, the hand used for reaching or the target that is reached? *Psychonomic Bulletin & Review*, *21*(1), 170–177. 10.3758/s13423-013-0488-y. 23918627 10.3758/s13423-013-0488-y

[CR16] Hoffmann, M. A., Pieczykolan, A., Koch, I., & Huestegge, L. (2019). Motor sources of dual-task interference: Evidence for effector-based prioritization in dual-task control. *Journal of Experimental Psychology: Human Perception and Performance*, *45*(10), 1355–1374. 10.1037/xhp0000677. 31343244 10.1037/xhp0000677

[CR17] Hommel, B. (2020). Dual-task performance: Theoretical analysis and an event-coding account. *Journal of Cognition*, *3*(1), 29. 10.5334/joc.114. 33043239 10.5334/joc.114 PMC7528664

[CR18] Huestegge, L. (2011). The role of saccades in multitasking: Towards an output-related view of eye movements. *Psychological Research Psychologische Forschung*, *75*(6), 452–465. 10.1007/s00426-011-0352-5. 21720887 10.1007/s00426-011-0352-5

[CR20] Huestegge, L., & Koch, I. (2009). Dual-task crosstalk between saccades and manual responses. *Journal of Experimental Psychology: Human Perception and Performance*, *35*(2), 352–362. 10.1037/a0013897. 19331493 10.1037/a0013897

[CR21] Huestegge, L., & Koch, I. (2010). Crossmodal action selection: Evidence from dual-task compatibility. *Memory & Cognition*, *38*(4), 493–501. 10.3758/MC.38.4.493. 20516229 10.3758/MC.38.4.493

[CR22] Huestegge, L., & Koch, I. (2014). When two actions are easier than one: How inhibitory control demands affect response processing. *Acta Psychologica*, *151*, 230–236. 10.1016/j.actpsy.2014.07.001. 25086224 10.1016/j.actpsy.2014.07.001

[CR24] Huestegge, L., & Strobach, T. (2021). Structuralist mental representation of dual-action demands: Evidence for compositional coding from dual tasks with low cross-task dimensional overlap. *Acta Psychologica*, *216*, 103298. 10.1016/j.actpsy.2021.103298. 33774503 10.1016/j.actpsy.2021.103298

[CR19] Huestegge, L., Herbort, O., Gosch, N., Kunde, W., & Pieczykolan, A. (2019). Free-choice saccades and their underlying determinants: Explorations of high-level voluntary oculomotor control. *Journal of Vision*, *19*(3), 14. 10.1167/19.3.14. 30924842 10.1167/19.3.14

[CR23] Huestegge, L., Pieczykolan, A., & Koch, I. (2023). A gestalt account of human behavior is supported by evidence from switching between single and dual actions. *Scientific Reports*, *13*(1). 10.1038/s41598-023-47788-0. 10.1038/s41598-023-47788-0 PMC10692127 38040736

[CR25] Koch, I., Poljac, E., Müller, H., & Kiesel, A. (2018). Cognitive structure, flexibility, and plasticity in human multitasking—An integrative review of dual-task and task-switching research. *Psychological Bulletin*, *144*(6), 557–583. 10.1037/bul0000144. 29517261 10.1037/bul0000144

[CR26] Kornblum, S., Hasbroucq, T., & Osman, A. (1990). Dimensional overlap: Cognitive basis for stimulus-response compatibility––A model and taxonomy. *Psychological Review*, *97*(2), 253–270. 10.1037/0033-295X.97.2.253. 2186425 10.1037/0033-295x.97.2.253

[CR27] Kürten, J., Raettig, T., Gutzeit, J., & Huestegge, L. (2022). Dual-action benefits: Global (action-inherent) and local (transient) sources of action prepotency underlying inhibition failures in multiple action control. *Psychological Research Psychologische Forschung*. 10.1007/s00426-022-01672-0. 35394557 10.1007/s00426-022-01672-0 PMC9928916

[CR28] Kürten, J., Raettig, T., Gutzeit, J., & Huestegge, L. (2023). Preparing for simultaneous action and inaction: Temporal dynamics and target levels of inhibitory control. *Journal of Experimental Psychology Human Perception and Performance*, *49*(7), 1068–1082. 10.1037/xhp0001126. 37227859 10.1037/xhp0001126

[CR29] Lakens, D., & Caldwell, A. (2023). *TOSTER: Two One-Sided Tests (TOST) Equivalence Testing* (0.8.0) [Computer software]. https://cran.r-project.org/web/packages/TOSTER/index.html.

[CR610] Lauzon, C., & Caffo, B. (2009). Easy multiplicity control in equivalence testing using two one-sided tests. *The American Statistician, 63*(2), 147–154. 10.1198/tast.2009.0029 PMC2800314 20046823

[CR30] Logan, G. D., & Cowan, W. B. (1984). On the ability to inhibit thought and action: A theory of an act of control. *Psychological Review*, *91*(3), 295–327. 10.1037/0033-295X.91.3.295. 10.1037/a0035230 24490789

[CR31] Logan, G. D., & Gordon, R. D. (2001). Executive control of visual attention in dual-task situations. *Psychological Review*, *108*(2), 393–434. 10.1037/0033-295X.108.2.393. 11381835 10.1037/0033-295x.108.2.393

[CR32] Lu, C., & Proctor, R. W. (1995). The influence of irrelevant location information on performance: A review of the Simon and spatial Stroop effects. *Psychonomic Bulletin & Review*, *2*(2), 174–207. 10.3758/BF03210959. 24203654 10.3758/BF03210959

[CR33] Luo, C., & Proctor, R. W. (2018). The location-, word-, and arrow-based Simon effects: An ex-gaussian analysis. *Memory & Cognition*, *46*(3), 497–506. 10.3758/s13421-017-0767-3. 29159679 10.3758/s13421-017-0767-3

[CR34] Lussier, M., Gagnon, C., & Bherer, L. (2012). An investigation of response and stimulus modality transfer effects after dual-task training in younger and older. *Frontiers in Human Neuroscience*, *6*. 10.3389/fnhum.2012.00129. 10.3389/fnhum.2012.00129 PMC3355323 22629239

[CR35] Mascaro, M., Battaglia-Mayer, A., Nasi, L., Amit, D. J., & Caminiti, R. (2003). The eye and the hand: Neural mechanisms and network models for oculomanual coordination in parietal cortex. *Cerebral Cortex*, *13*(12), 1276–1286. 10.1093/cercor/bhg075. 14615294 10.1093/cercor/bhg075

[CR36] Matzke, D., Verbruggen, F., & Logan, G. D. (2018). The stop-signal paradigm. In J. T. Wixted (Ed.), *Stevens’ handbook of experimental psychology and cognitive neuroscience*. John Wiley & Sons, Inc. 10.1002/9781119170174.epcn510.

[CR37] May, J. G., Kennedy, R. S., Williams, M. C., Dunlap, W. P., & Brannan, J. R. (1990). Eye movement indices of mental workload. *Acta Psychologica*, *75*(1), 75–89. 10.1016/0001-6918(90)90067-P. 2260494 10.1016/0001-6918(90)90067-p

[CR38] Meeter, M., Van der Stigchel, S., & Theeuwes, J. (2010). A competitive integration model of exogenous and endogenous eye movements. *Biological Cybernetics*, *102*(4), 271–291. 10.1007/s00422-010-0365-y. 20162429 10.1007/s00422-010-0365-y

[CR39] Meyer, D. E., & Kieras, D. E. (1997). A computational theory of executive cognitive processes and multiple-task performance: Part 2. Accounts of psychological refractory-period phenomena. *Psychological Review*, *104*(4), 749–791. 10.1037/0033-295X.104.4.749. 10.1037/0033-295x.104.1.3 9009880

[CR40] Miles, J. D., & Proctor, R. W. (2012). Correlations between spatial compatibility effects: Are arrows more like locations or words? *Psychological Research Psychologische Forschung*, *76*(6), 777–791. 10.1007/s00426-011-0378-8. 21909980 10.1007/s00426-011-0378-8

[CR161] Miller, J. (2006). Backward crosstalk effects in psychological refractory period paradigms: Effects of second-task response types on first-task response latencies. *Psychological Research, 70*(6), 484–493. 10.1007/s00426-005-0011-9. 10.1007/s00426-005-0011-9 16237555

[CR41] Moeller, B., & Frings, C. (2019a). From simple to complex actions: Response–response bindings as a new approach to action sequences. *Journal of Experimental Psychology: General*, *148*(1), 174–183. 10.1037/xge0000483. 30211579 10.1037/xge0000483

[CR42] Moeller, B., & Frings, C. (2019b). Lost time: Bindings do not represent temporal order information. *Psychonomic Bulletin & Review*, *26*(1), 325–331. 10.3758/s13423-018-1493-y. 29869024 10.3758/s13423-018-1493-y

[CR43] Moeller, B., & Frings, C. (2019c). Response–response binding across effector-set switches. *Psychonomic Bulletin & Review*, *26*(6), 1974–1979. 10.3758/s13423-019-01669-8. 31654376 10.3758/s13423-019-01669-8

[CR44] Müller, H. J., & Rabbitt, P. M. (1989). Reflexive and voluntary orienting of visual attention: Time course of activation and resistance to interruption. *Journal of Experimental Psychology: Human Perception and Performance*, *15*(2), 315–330. 10.1037/0096-1523.15.2.315. 2525601 10.1037//0096-1523.15.2.315

[CR45] Navon, D., & Miller, J. (1987). Role of outcome conflict in dual-task interference. *Journal of Experimental Psychology: Human Perception and Performance*, *13*(3), 435–448. 10.1037/0096-1523.13.3.435. 2958592 10.1037//0096-1523.13.3.435

[CR46] Navon, D., & Miller, J. (2002). Queuing or sharing? A critical evaluation of the single-bottleneck notion. *Cognitive Psychology*, *44*(3), 193–251. 10.1006/cogp.2001.0767. 11971632 10.1006/cogp.2001.0767

[CR47] Pashler, H. (1994). Dual-task interference in simple tasks: Data and theory. *Psychological Bulletin*, *116*(2), 220–244. 10.1037/0033-2909.116.2.220. 7972591 10.1037/0033-2909.116.2.220

[CR48] Pieczykolan, A., & Huestegge, L. (2014). Oculomotor dominance in multitasking: Mechanisms of conflict resolution in cross-modal action. *Journal of Vision*, *14*(13), 18–18. 10.1167/14.13.18. 25406163 10.1167/14.13.18

[CR49] Posner, M. I. (1980). Orienting of attention. *The Quarterly Journal of Experimental Psychology*, *32*, 3–25. 10.1080/00335558008248231. 7367577 10.1080/00335558008248231

[CR50] Posner, M. I. (2016). Orienting of attention: Then and now. *The Quarterly Journal of Experimental Psychology*, *69*(10), 1864–1875. 10.1080/17470218.2014.937446. 25176352 10.1080/17470218.2014.937446 PMC4345129

[CR51] Raettig, T., & Huestegge, L. (2018). The hard work of doing nothing: Accounting for inhibitory costs during multiple action control. *Attention Perception & Psychophysics*, *80*(7), 1660–1666. 10.3758/s13414-018-1577-9. 10.3758/s13414-018-1577-9 30069681

[CR52] Raettig, T., & Huestegge, L. (2021). Representing action in terms of what not to do: Evidence for inhibitory coding during multiple action control. *Journal of Experimental Psychology: Human Perception and Performance*, *47*(9), 1253–1273. 10.1037/xhp0000943. 34694854 10.1037/xhp0000943

[CR53] Raettig, T., & Huestegge, L. (2023). Explaining dual-action benefits: Inhibitory control and redundancy gains as complementary mechanisms. *Journal of Experimental Psychology Learning Memory and Cognition*. 10.1037/xlm0001231. 37079846 10.1037/xlm0001231

[CR54] Remington, R. W. (1980). Attention and saccadic eye movements. *Journal of Experimental Psychology Human Perception and Performance*, *6*(4), 726–744. 10.1037//0096-1523.6.4.726. 10.1037//0096-1523.6.4.726 6449540

[CR55] Ridderinkhof, K. R., van den Wildenberg, W. P. M., & Brass, M. (2014). Don׳t versus won׳t: Principles, mechanisms, and intention in action inhibition. *Neuropsychologia*, *65*, 255–262. 10.1016/j.neuropsychologia.2014.09.005. 25218168 10.1016/j.neuropsychologia.2014.09.005

[CR56] Rosenbaum, D. A. (1985). Motor programming: A review and scheduling theory. In H. Heuer, U. Kleinbeck, & K.-H. Schmidt (Eds.), *Motor Behavior* (pp. 1–33). Springer Berlin Heidelberg. 10.1007/978-3-642-69749-4_1.

[CR57] Schumacher, E. H., Seymour, T. L., Glass, J. M., Fencsik, D. E., Lauber, E. J., Kieras, D. E., & Meyer, D. E. (2001). Virtually perfect time sharing in dual-task performance: Uncorking the central cognitive bottleneck. *Psychological Science*, *12*(2), 101–108. 10.1111/1467-9280.00318. 11340917 10.1111/1467-9280.00318

[CR58] Spering, M. (2022). Eye movements as a window into decision-making. *Annual Review of Vision Science*, *8*(8, 2022), 427–448. 10.1146/annurev-vision-100720-125029. 35676097 10.1146/annurev-vision-100720-125029

[CR59] Strobach, T., & Huestegge, L. (2021). Structuralist mental representation of dual-action demands: Mechanisms of improved dual-task performance after practice in older adults. *Experimental Aging Research*, *47*(2), 109–130. 10.1080/0361073X.2021.1873053. 33446078 10.1080/0361073X.2021.1873053

[CR60] Stuyven, E., Van der Goten, K., Vandierendonck, A., Claeys, K., & Crevits, L. (2000). The effect of cognitive load on saccadic eye movements. *Acta Psychologica*, *104*(1), 69–85. 10.1016/S0001-6918(99)00054-2. 10769940 10.1016/s0001-6918(99)00054-2

[CR61] Verbruggen, F., & Logan, G. D. (2015). Evidence for capacity sharing when stopping. *Cognition*, *142*, 81–95. 10.1016/j.cognition.2015.05.014. 26036922 10.1016/j.cognition.2015.05.014 PMC4787292

