# Supplementary material for: Erroneous saccade co-execution during manual action control is independent of oculomotor stimulus-response translation ease
Source: Psychol Res. 2024 Jul 30;88(8):2376–88. doi: 10.1007/s00426-024-01989-y (PMC11522101; doi:10.1007/s00426-024-01989-y)
Supplement: Supplementary file 1 — Supplementary Material 1 [file 426_2024_1989_MOESM1_ESM.docx]

Supplement to: Erroneous saccade co-execution during manual action control is independent of oculomotor stimulus-response translation ease

Jens Kürten^1^, Tim Raettig^1^, & Lynn Huestegge^1^

^1^ University of Wuerzburg

Author note

Jens Kürten, Department of Psychology, University of Würzburg (<https://orcid.org/0000-0002-5903-1341>);

Tim Raettig, Department of Psychology, University of Würzburg;

Julian Gutzeit, Department of Psychology, University of Würzburg;

Lynn Huestegge, Department of Psychology, University of Würzburg.

The authors made the following contributions. Jens Kürten: Conceptualization, Writing - Original Draft Preparation, Writing - Review & Editing; Tim Raettig: Writing - Review & Editing; Lynn Huestegge: Writing - Review & Editing, Supervision.

Correspondence concerning this article should be addressed to Jens Kürten, Roentgenring 11, 97070 Wuerzburg. E-mail: [jens.kuerten@uni-wuerzburg.de](mailto:jens.kuerten@uni-wuerzburg.de)

**Abstract**

Recent multiple action control studies have demonstrated difficulties with single-action (vs. dual-action) execution when accompanied by the requirement to inhibit a prepotent additional response (e.g., a highly automatic eye movement). Such a dual-action performance benefit is typically characterized by frequent false-positive executions of the currently unwarranted response. Here, we investigated whether the frequency of false-positive saccades is affected by the ease of translating a stimulus into a spatial oculomotor response (S-R translation ease): Is it harder to inhibit a saccade that is more automatically triggered via the stimulus? Participants switched on a trial-by-trial basis between executing a single saccade, a single manual button press, and a saccadic-manual dual action in response to a single visual stimulus. Importantly, we employed three different stimulus modes that varied in oculomotor S-R translation ease (peripheral square > central arrow > central shape). The hierarchy of S-R translation ease was reflected by increasing saccade and manual reaction times. Critically, however, the frequency of false-positive saccades in single manual trials was not substantially affected by the stimulus mode. Our results rule out explanations related to limited capacity sharing (between inhibitory control and S-R translation demands) as well as accounts related to the time available for the completion of saccade inhibition. Instead, the findings suggest that the erroneous co-activation of the oculomotor system was elicited by the mere execution of a (frequently associated) manual response (action-based co-activation).

*Keywords:* multiple action control, peripheral stimuli, symbolic stimuli, automaticity, dual-action benefits

**Supplement to: Erroneous saccade co-execution during manual action control is independent of oculomotor stimulus-response translation ease**

Table S1 contains descriptive summary statistics of reaction times (RTs) and error rates (ERs) of both the saccade and manual responses as a function of action demand (dual vs. single saccade vs. single manual) and stimulus mode (peripheral square vs. central arrow vs. central shape) corresponding to the data plotted in Figures 2 and 3 in the manuscript (page 18 and page 20, respectively). Figure S1 displays ERs of saccade and manual responses as a function of error type (false-direction vs. false-negative vs. false-positive) and stimulus mode (instead of as a function of action demand and stimulus mode) and is referenced in the manuscript on page 23. Table S2 and Figure S2 correspond to an exploratory analysis of saccade amplitudes in correct saccades in single-saccade and dual-action trials and false-positive saccades in single-manual trials across stimulus modes. False-positive saccade amplitudes were (consistently) significantly smaller than correct saccade amplitudes indicating competition between activation and inhibition of the saccade system (referenced on page 25 of the manuscript). Figure S3 shows the percentage of dual-action trials and single-manual trials with a false-positive saccade in which the saccade and the manual response went in the same direction (referenced on page 28 of the manuscript). Finally, Table S3 and Figure S4 display mean correlations between saccade and manual RTs in dual-action trials and single-manual trials with a false-positive saccade across the different stimulus modes and the inter-response intervals between saccade and manual responses in these trials. The patterns observed indicate a rather independent execution of false-positive saccades and (the actually required) manual responses in single-manual trials across the three stimulus modes (referenced on pages 28 – 29 of the manuscript).

**Table S1**
*Descriptive summary statistics*

| Stimulus Mode | Action Demand | Modality | Mean RT [ms] | *SE_M_* RT | Mean ER [%] | *SE_M_* ER |
| --- | --- | --- | --- | --- | --- | --- |
| Peripheral Square | Dual | Saccade | 226 | 7 | 3.93 | 0.75 |
|  |  | Manual | 423 | 12 | 2.31 | 1.11 |
|  | Single Saccade | Saccade | 232 | 8 | 4.31 | 0.74 |
|  |  | Manual | NA | NA | 3.29 | 0.60 |
|  | Single Manual | Saccade | NA | NA | 18.96 | 1.90 |
|  |  | Manual | 393 | 9 | 4.46 | 0.58 |
| Central Arrow | Dual | Saccade | 317 | 6 | 3.37 | 0.66 |
|  |  | Manual | 500 | 13 | 2.54 | 1.00 |
|  | Single Saccade | Saccade | 311 | 6 | 4.07 | 0.79 |
|  |  | Manual | NA | NA | 2.26 | 0.40 |
|  | Single Manual | Saccade | NA | NA | 16.07 | 1.61 |
|  |  | Manual | 445 | 8 | 5.13 | 0.73 |
| Central Shape | Dual | Saccade | 403 | 8 | 13.78 | 1.39 |
|  |  | Manual | 593 | 11 | 9.83 | 1.58 |
|  | Single Saccade | Saccade | 400 | 9 | 15.48 | 1.76 |
|  |  | Manual | NA | NA | 4.38 | 0.53 |
|  | Single Manual | Saccade | NA | NA | 19.46 | 1.98 |
|  |  | Manual | 549 | 10 | 14.90 | 1.57 |

*Note*. RT = reaction time; *SE_M_* = standard error of the mean; ER = error rate.

**Table S2**
*ANOVA results of saccade amplitudes as a function of Stimulus Mode and Action Demand*

| Effect | $\hat{\eta}_{p}^{2}$ | $F$ | ${df}^{\mathrm{GG}}$ | ${df}_{\mathrm{res}}^{\mathrm{GG}}$ | $p$ |
| --- | --- | --- | --- | --- | --- |
| Action Demand | .555 | 57.26 | 1.32 | 60.65 | < .001 |
| Stimulus Mode | .031 | 1.46 | 1.99 | 91.34 | .237 |
| Action Demand $\times$ Stimulus Mode | .145 | 7.79 | 2.20 | 101.17 | < .001 |

*Note*. $\hat{\eta}_{p}^{2}$= partial eta squared; ${df}^{\mathrm{GG}}$ = Greenhouse-Geisser adjusted degrees of freedom.

**Table S3**

*Correlations between saccade and manual RTs as a function of stimulus mode and action demand*

| Stimulus Mode | Action Demand | Mean *n* trials | *r_min_* | *r_max_* | Proportion significant | Mean *r* |
| --- | --- | --- | --- | --- | --- | --- |
| Peripheral Square | Dual | 53.56 | .149 | .861 | 87.50 | .51 |
|  | Single Manual | 8.67 | -.741 | .986 | 13.64 | .21 |
| Central Arrow | Dual | 54.73 | .011 | .791 | 75.00 | .47 |
|  | Single Manual | 7.40 | -.987 | .999 | 16.22 | .22 |
| Central Shape | Dual | 48.23 | .252 | .903 | 91.67 | .67 |
|  | Single Manual | 6.29 | -1.00 | .916 | 10.81 | .28 |

*Note*. Mean *n* trials = average number of trials in condition with both a saccade and manual RT, *r_min_* = minimum intraindividual Pearson correlation coefficient *r_max_* = maximum intraindividual Pearson correlation coefficient, Mean *r* = average Pearson correlation coefficient.

**Figure S1**
*Error rates as a function of stimulus mode and error type*
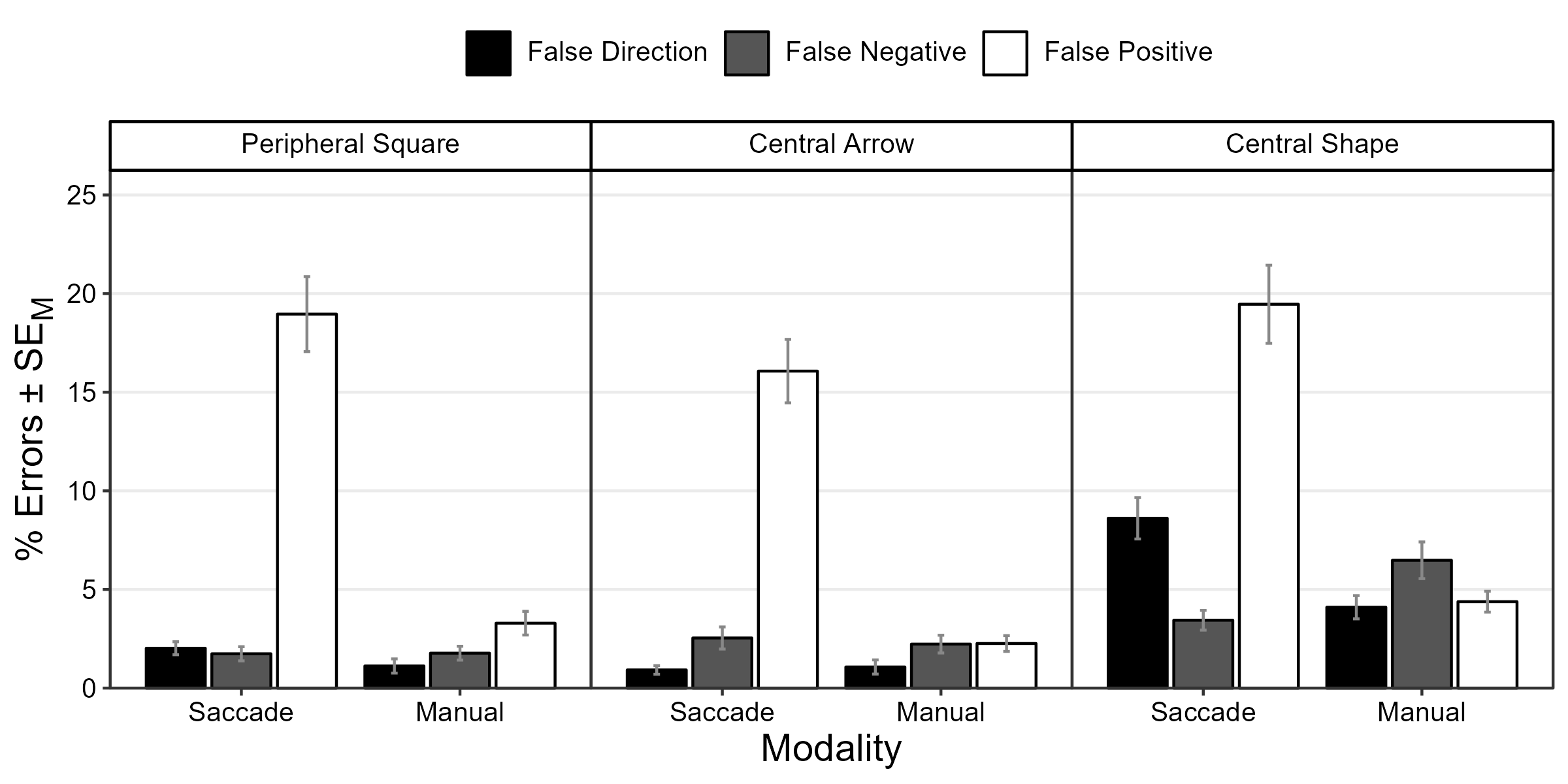
*Note*. ERs (%) of both response modalities (saccade, manual) as a function of stimulus mode (peripheral square, central arrow, central shape) and error type (false direction, false negative, false positive, false positive and false direction), calculated from all *trial types* in which a certain *error type* could occur. False-positive error rates are based on 60 trials per modality and stimulus mode, false-negative error rates are based on 120 trials per modality and stimulus mode, and false-direction errors are based on 180 trials per modality and stimulus mode. Error rates depicted here thus do not directly correspond to the error rates depicted in Figure 3 in the manuscript due to different numbers of trials considered. Error bars represent the standard error of the mean (SE_M_).

**Figure S2***Saccade amplitude as a function stimulus mode and action demand*


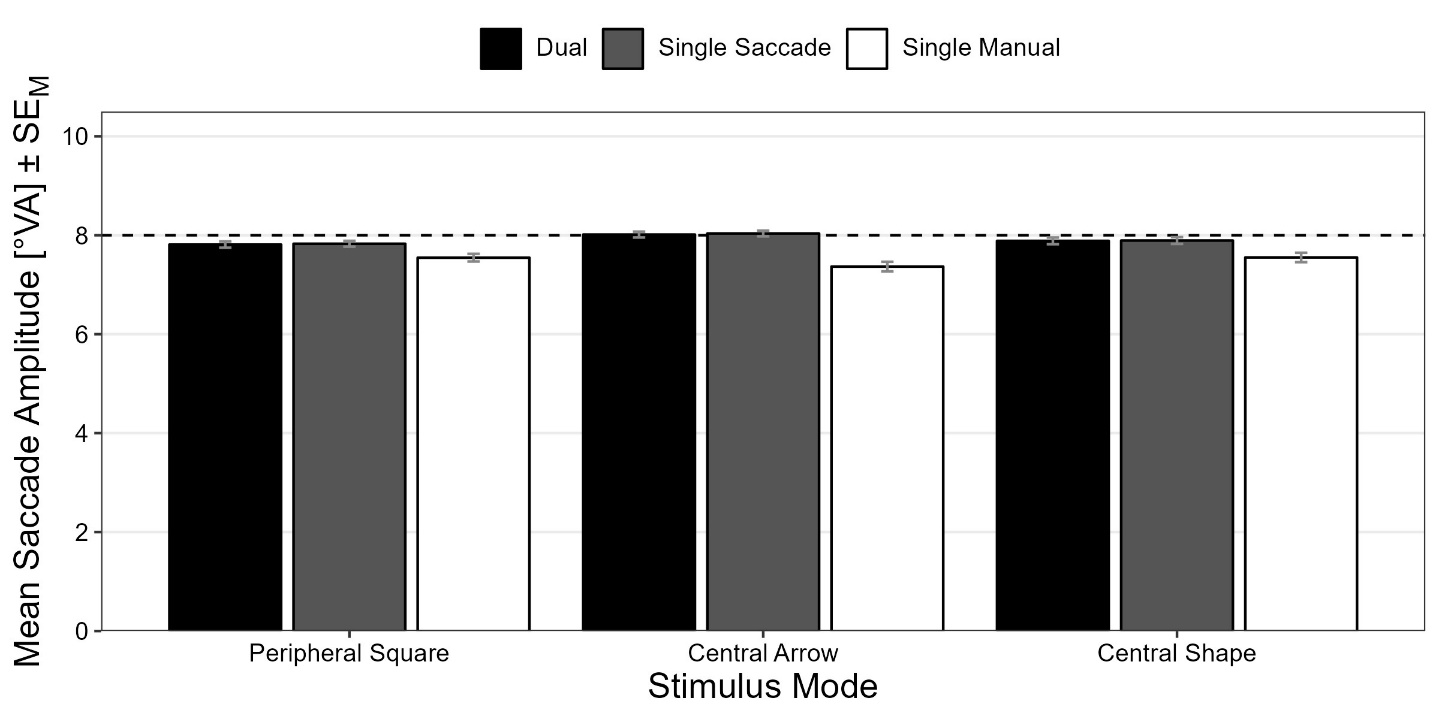


*Note.* Saccade amplitudes averaged across left and right target positions. Results are based on saccades in the correct (as indicated by the stimulus) direction only. Saccades in Single Manual are false-positives. The dashed horizontal line indicates the distance of the target from the screen center. Error bars represent the standard error of the mean (SE_M_).

**Figure S3***Percentage of warranted and erroneous dual actions with both component actions in the same direction*
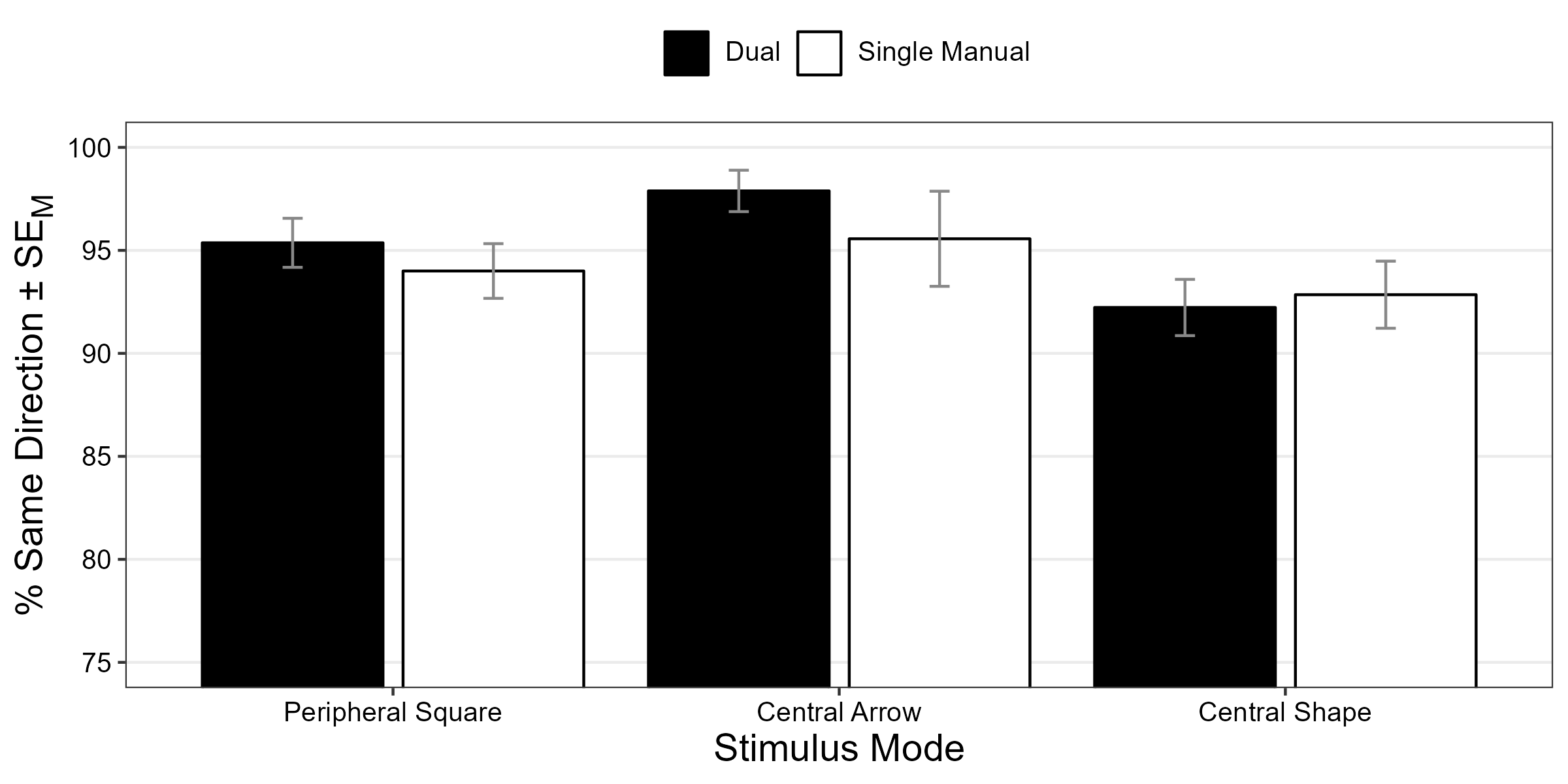
*Note.* Percentage of dual-action trials and single manual trials with the (false-positive) saccade and a manual response with both actions carried out in the same direction (both left or both right). The mean trial count in the single manual condition was 9.98 (SD = 7.65), 8.30 (SD = 6.41), and 8.52 (SD = 7.21) with the peripheral square, the central arrow, and the central shape respectively. Error bars represent the standard error of the mean (SE_M_).

**Figure S4***Distribution of inter-response intervals (IRIs) as a function of Stimulus Mode and Action Demand*
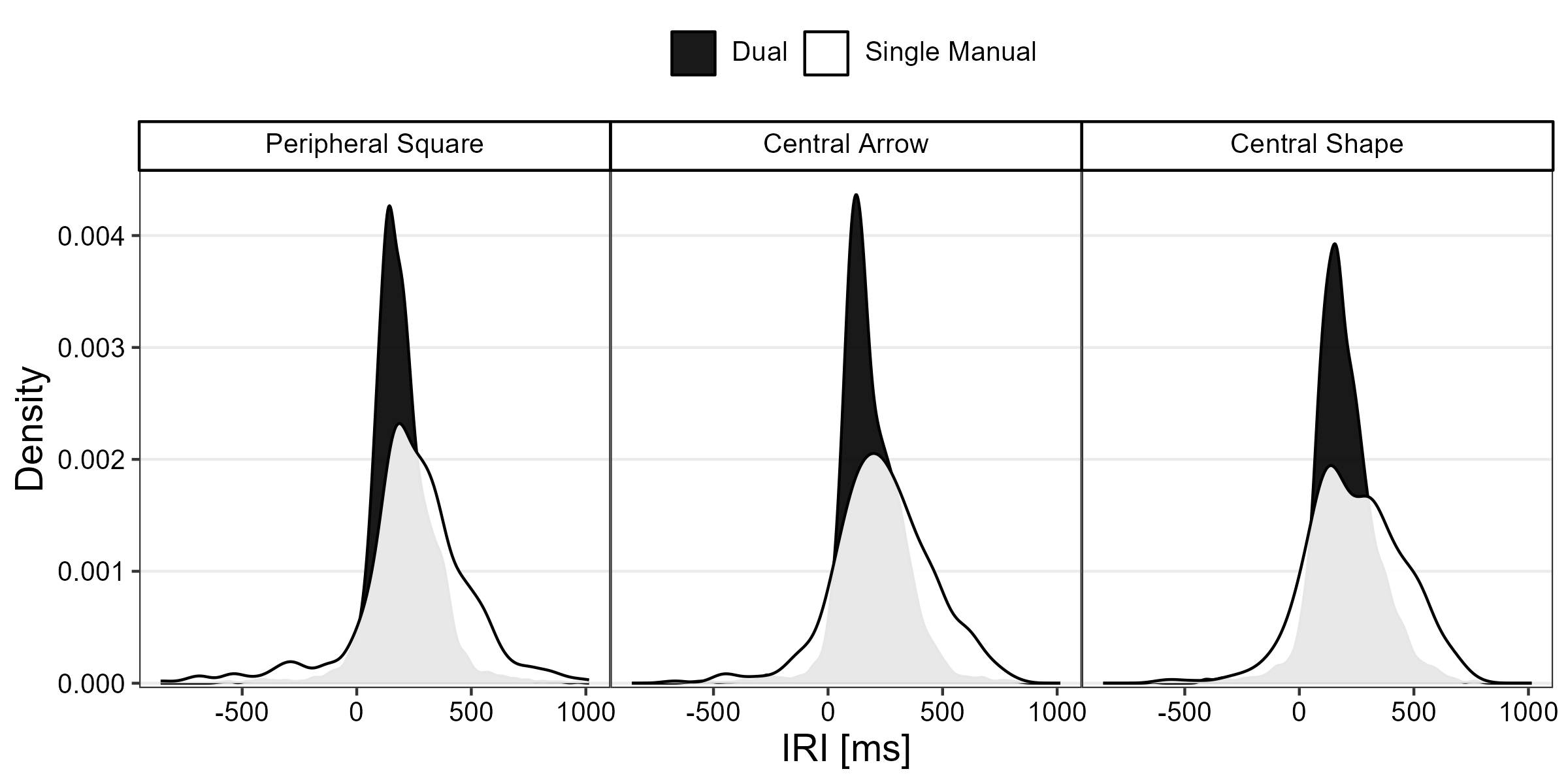
*Note.* Distribution of inter-response intervals (IRIs) in ms in dual-action trials (black-filled curve), and in single manual trials with a false-positive saccade and a manual response (white-filled curve) pooled across all 48 participants. Positive values of IRI indicate that the saccade was executed before the key press, negative values indicate that the key press was executed before the saccade.
